# Impact of a brief diabetes education on teachers’ diabetes-related knowledge, attitude, and confidence: a nonrandomized school-based intervention study

**DOI:** 10.3389/fpubh.2026.1747185

**Published:** 2026-03-25

**Authors:** Maria Dora Horváth, Zsanett Tesch, Norbert Buzás, Orsolya Papp-Zipernovszky

**Affiliations:** 1 Institute of Psychology, Department of Personality, Clinical and Health Psychology, University of Szeged, Szeged, Hungary; 2 Albert Szent-Györgyi Medical School, Department of Health Economics, University of Szeged, Szeged, Hungary; 3 Department of Theoretical Health Sciences and Health Management, Faculty of Health Sciences and Social Studies, University of Szeged, Szeged, Hungary; 4 Institute of Psychology, Department of Personality and Health Psychology, Faculty of Education and Psychology, Eötvös Loránd University, Budapest, Hungary; 5 Heart and Cardiovascular Centre, Faculty of Medicine, Semmelweis University, Budapest, Hungary

**Keywords:** attitude, confidence in diabetes care, diabetes knowledge, school, teachers, type 1 diabetes

## Abstract

**Background and aims:**

The involvement of kindergarten and school teachers is crucial for ensuring safe diabetes management in educational settings for children with type 1 diabetes. This nonrandomized, school-based intervention study aims to assess the effectiveness of a one-hour diabetes education program, delivered either online or in person, on teachers’ knowledge of diabetes, attitudes toward diabetes, and confidence in diabetes care.

**Methods:**

A one-hour diabetes education session led by a diabetes educator included both theoretical and practical modules on managing type 1 diabetes. Participants completed the Diabetes Knowledge Test 2, the Diabetes Attitude Survey 3, the School Personnel Diabetes Attitude Scale, and the Confidence in Diabetes Care scale before, immediately after, and 1 month following the intervention. The session was delivered to 60 educators (24 in person, 36 online; mean age = 43.72 years, SD = 9.48; 56 female, 4 male). Participants were assigned to conditions based on availability rather than randomization.

**Results:**

The education program significantly improved diabetes knowledge among all participants, and this improvement was maintained at the one-month follow-up. Confidence in diabetes care also increased after the intervention; however, confidence declined at follow-up in the online group compared to the in-person group.

**Conclusion:**

A brief, one-hour diabetes education session is enough to achieve lasting improvements in teachers’ diabetes knowledge and confidence. In-person teaching seems more effective at maintaining confidence over time, though online education remains a practical option. Incorporating structured diabetes education into teacher training programs could help create safer, more supportive school environments for children with type 1 diabetes.

## 1 Introduction

Recent European data show a significant increase in the incidence of type 1 diabetes among children and adolescents over recent decades, with incidence rates nearly doubling between 1994–2003 and 2013–2022 (1). In Hungary, where the present study was conducted, national statistics indicate that nearly 5,000 children live with type 1 diabetes (2). This ongoing rise in incidence suggests a growing number of children living with type 1 diabetes, highlighting the increasing importance of diabetes support in everyday environments such as schools.

Beyond its medical aspects, the experience and management of type 1 diabetes in childhood are strongly influenced by the child’s immediate social environment, including family members, educational settings, and healthcare systems. From a social-ecological perspective, schools are a key microsystem in children’s daily lives, where teachers’ preparedness and attitudes can directly affect both psychosocial well-being and diabetes management outcomes (3–5). The availability of school-based healthcare support varies greatly across countries, and many educational settings lack consistent on-site nursing care for children with chronic conditions (6, 7). The MOCHA project reported significant international differences in school nurse availability, with about 1.4 school nurses per 1,000 students in countries like Norway and Estonia, and lower coverage in others, including Iceland (7). In Hungary, school health services are organized within the primary healthcare system rather than through a dedicated, school-based nursing model (8); as a result, children with T1D often lack continuous healthcare support at schools and kindergartens. According to Pansier and Schulz (9), children with type 1 diabetes and their parents often depend on school staff for support with diabetes management in educational settings. Challenges usually stem from insufficiently trained personnel, the absence of school nurses, or unclear diabetes care policies, which can lead to absenteeism, psychological distress, reduced academic performance, and lower quality of life (9). Therefore, providing school personnel with diabetes-related knowledge and confidence holds significant practical importance, as it may lead to safer diabetes care, better psychological adjustment, and increased participation in education for children living with type 1 diabetes (10, 11). Teachers play a crucial role not only in supporting children with diabetes management tasks but also in the psychological processes involved in living with T1D, such as accepting their diagnosis and helping peers understand their condition (12).

Several studies have examined teachers’ perceptions and attitudes toward diabetes care (13–16). Holmström et al. (17) explored school personnel’s experiences caring for youth with T1D through interviews. They described their experience as “being facilitators in a challenging context” (p. 116), feeling uncertain and overwhelmed by constant, unclear responsibilities. Teachers had to find their own ways to cope with these challenges, support self care, and build trusting relationships with students and their parents. Luque-Vara et al. (18) used questionnaires to assess perceptions and attitudes toward diabetes care among teachers. Half of the respondents said their schools were unprepared to handle diabetes-related situations; only 4.8% had attended a diabetes education program, 29.9% had witnessed a hypoglycemic episode at school, and 44. 6% were willing to administer glucagon to a student. A Spanish study found that while 43% of teachers had taught children with diabetes, only 0. 0.8% had received specific training (15). Many teachers lack knowledge about the differences between type 1 and type 2 diabetes, cannot recognize typical symptoms like nausea, and hold various misconceptions about diabetes (19). Both Tolbert (20), in a review, and the American Diabetes Association (21) underscored the need to educate school staff. Among non-diabetic individuals, greater diabetes knowledge and higher education levels are associated with more positive attitudes toward diabetes (13, 22). Consistent with these findings, Tannous et al. (23) reported that teachers demonstrated moderate levels of diabetes knowledge and generally favorable attitudes toward students with diabetes. Positive attitudes can foster effective diabetes management; therefore, attitudes through education may help ensure that children with diabetes receive proper care at school. These findings suggest that diabetes knowledge and attitudes are interconnected (13, 22), and that educational interventions could promote more positive attitudes.

The KiDS and Diabetes in Schools program (24) aims to improve understanding and management of diabetes in schools. The part of the program focusing on type 1 diabetes highlights students’ needs at school and includes a simple diabetes management plan. Bechara et al. (19) evaluated its effectiveness and found that 56% of school staff had not previously heard of diabetes in a school setting. However, after training, 82% reported changed attitudes, 32% felt more willing to participate in diabetes care, and 38% felt confident approaching families about diabetes management.

Interactive e-learning programs have also demonstrated promise in diabetes education for school personnel. Taha et al. (25) evaluated an online program that combined diabetes knowledge-based content with skills development and observed significant improvements in both diabetes knowledge and confidence, with effects maintained at long-term follow-up. Gutierrez (26) examined the “Diabetes Care at School: Bridging the Gap” program and reported notable gains in diabetes knowledge and self-efficacy among school staff, especially among non-medical personnel, whose post-training scores nearly matched those of previously trained participants. Dixe et al. (27) used two 3-h face-to-face sessions to cover theoretical and practical modules of diabetes management. Following training, there were significant increases in both knowledge and confidence scores. Zimmerman et al. (28) evaluated a virtual diabetes education program and found high perceived benefits and improved preparedness among participating school personnel. A quasi-experimental intervention in Turkey demonstrated that a brief, structured diabetes education significantly enhanced teachers’ diabetes-related knowledge, self-efficacy, and applied skills in a school setting (29). Similarly, a study in Morocco involving over 300 teachers reported substantial improvements in knowledge of type 1 diabetes following targeted education (30). Bassi et al. (31) assessed the effectiveness of an online diabetes training program for school staff in Italy that combined theoretical and practical modules. Trained participants demonstrated greater diabetes-related knowledge and greater confidence in managing both routine care and emergency situations than untrained staff, supporting the effectiveness of online education in improving school personnel’s preparedness.

While several studies have shown that diabetes education programs, delivered either face-to-face or online, can improve school personnel’s diabetes-related knowledge and confidence, these interventions usually involve longer or multi-component programs. There is comparatively little evidence on whether a brief, standardized, single-session education program is sufficient to produce measurable and lasting improvements. In this study, we aimed to evaluate a short, one-hour diabetes education intervention delivered in both in-person and online formats. The intervention was pragmatic and not explicitly based on theory. The study’s research question is whether participation in the education program leads to measurable changes in these outcomes immediately after the intervention and at one-month follow-up. Based on the previous findings, we hypothesize that participation results in (1) increased diabetes-related knowledge and (2) increased confidence in diabetes care. Given the mixed findings in the literature regarding attitude change following brief educational interventions, changes in diabetes-related attitudes are examined exploratorily. Furthermore, we hypothesize that the magnitude of these changes is greater following in-person education compared to online delivery.

## 2 Methods

### 2.1 Study design

The reporting of this study adhered to the TREND (Transparent Reporting of Evaluations with Nonrandomized Designs) statement (32). A convenience sampling method was employed by directly contacting school and kindergarten principals. Additionally, we recruited participants through the regional school psychology network. Participants did not receive any financial compensation for participating. During recruitment, we highlighted a free opportunity to receive diabetes education. Eligible participants were practicing teachers working in public schools or kindergartens. The study focused on teachers in direct teaching roles; school principals and administrative staff were not included. Participants had to be employed full-time as teachers at the time of the study. There were no restrictions based on age or previous experience with students living with type 1 diabetes, as the goal was to improve general preparedness. All participants provided written informed consent prior to participating. The final sample consisted of 60 teachers from five public schools and two public kindergartens. The sample size was determined based on feasibility and participant availability within the study period. All teachers who met the criteria and were available during recruitment were included.

In Hungary, where the study was conducted, continuous diabetes care was generally unavailable in public educational settings. Although school nurses and physicians are officially employed, they are usually not present daily; only one participant worked in a setting that offered continuous on-site healthcare. Of the total sample, 24 participants received diabetes education in person, and 36 received it online. The unit of assignment was the individual teacher. Allocation to in-person or online groups was determined by school-level feasibility and participant availability. Due to the pragmatic design, no formal randomization or matching was used. Detailed demographic information of the participants is provided in Table 1. Baseline differences between the in-person and online groups in age and years of experience were assessed using independent-samples t-tests. The analyses showed no significant baseline differences between the two groups. Age did not differ significantly between groups, *t*(58) = 0.87, *p* = 0.390, nor did years of teaching experience, *t*(57) = 0.78, *p* = 0.442 (see Table 1). Levene’s tests confirmed that the assumption of homogeneity of variances was met for both variables (all *p* > 0.23).

**Table 1:** Participants’ characteristics (Age and years spent working as a teacher are shown in mean values together with the standard deviations).

Sociodemographic factors	All participants (*n* = 60)	In-person group (*n* = 24)	Online group (*n* = 36)	*p* (baseline differences)
Gender				
Male	4	3	1	
Female	56	21	35	
Age they work with				
Kindergarten (children aged 3–5 years)	37	10	27	
Primary school (children aged 6–13 years)	23	14	9	
Taking part of managing diabetes in school				
Yes, handling management tasks	39	13	26	
Being attentive of symptoms and notifying parents	11	2	9	
No	10	9	1	
Age in years Years spent working as a teacher	43.72 (9.48) 16.9 (11.5)	44.6 (9.93) 20.6 (11.4)	43.1 (11.4) 14.5 (11.1)	0.390 0.442

Participants completed a pretest before the diabetes education session, a post-test immediately afterward, and a retest 30 days later. To maintain anonymity while enabling data linkage across measurement points, participants provided a self-generated code name. In the in-person condition, questionnaires were completed on paper and collected immediately, whereas in the online condition, participants completed the surveys independently via a secure digital platform. They were informed that the questionnaire could only be completed once without interruption and that three separate assessments (pretest, post-test, and retest) would be conducted. All participants gave informed consent before participating. The research was approved by the Human Investigation Review Board at the University of Szeged Albert Szent-Gyorgyi Clinical Center (Ethics Opinion 199/2019-SZTE).

### 2.2 Diabetes education

**Figure 1:**
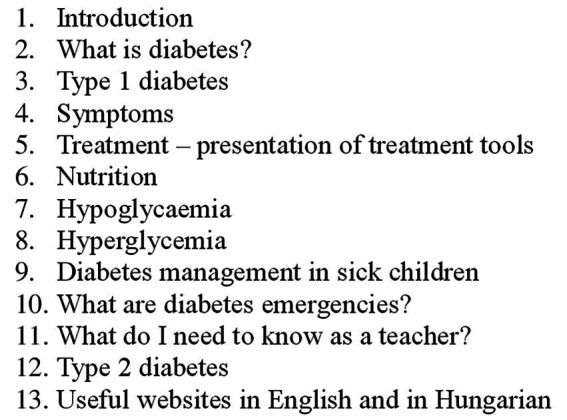
Main themes of the diabetes education.

We used two materials to develop the diabetes education: the “KiDS and Diabetes in Schools” package (24), compiled by the International Diabetes Federation (IDF), and the guide titled “What is type 1 diabetes? A guide for parents of newly diagnosed children” created by Novo Nordisk (33). The themes of diabetes education are shown in Figure 1.

**Figure 2:**
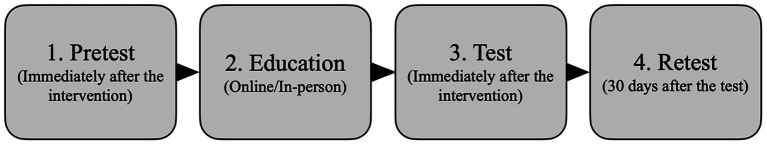
The process of the intervention.

The education session was led by a professional diabetes educator. It was held in person in the classrooms of the schools where the participants were enrolled, with groups of 6–12 members. The PowerPoint presentation included a physical demonstration of the treatment devices—such as the blood glucose meter, sensor, pen, and insulin pump—and a brief explanation of how they work. The session lasted 1 hour. Afterward, participants had the opportunity to ask any questions about the topic. Participants were asked to complete the questionnaire immediately before and after the education. The process of the educational intervention and knowledge level assessment is shown in Figure 2.

The online training was provided through a video hosted on an online platform. The video displayed a PowerPoint presentation accompanied by the diabetes educator’s voice narration. Participants could view the video at their convenience; they were instructed to complete the questionnaire both before and after watching it. A step-by-step guide for completing the questionnaires was provided. No formal monitoring was conducted to ensure participants fully engaged with the online educational material, and no incentives were offered for participation. The group of online participants had the option to join a Zoom video chat with the diabetes educator to ask questions; however, this option was seldom used, with only four participants asking questions. Recruitment and data collection spanned 6 months, with educational sessions delivered continuously as participants became available. Due to the nature of the intervention, blinding participants and educators was not feasible. Outcome measures depended on self-reported questionnaires.

A total of 60 teachers participated in the study, all meeting the eligibility criteria and providing informed consent. Participants were assigned to either the in-person (*n* = 24) or online (*n* = 36) education condition based on availability. All received their assigned intervention and completed the post-intervention and one-month follow-up assessments. One participant was excluded from the final analysis due to excessive missing data, leaving 59 participants in the final sample. No major protocol deviations occurred during the study.

### 2.3 Questionnaires

At the time of the study, no validated Hungarian instrument existed to assess teachers’ knowledge and attitudes regarding diabetes. Moreover, international studies often use study-specific measures with limited psychometric validation in teacher populations. Therefore, we adapted established instruments from the literature and assessed their psychometric properties in our sample. We based our measurements on the items of the Diabetes Attitude Scale (DAS-3) (34), a diabetes attitude scale for teachers developed by Tannous et al. (23), the confidence in diabetes care measure by Van der Ven et al. (35), and the Diabetes Knowledge Test 2 (DKT-2) (36). Where no validated Hungarian version was available, we performed a standard back translation process. After adapting and translating the items, we conducted exploratory factor analyses to identify the most suitable factor structures, and only the subscales and items with adequate psychometric properties were included in further analyses. Details of item selection and adaptation for each questionnaire are provided below, along with the factor analysis results in the appendices.

### 2.4 Diabetes knowledge test 2

To assess diabetes knowledge, a modified version of the Hungarian Diabetes Knowledge Test (DKT 2) (37); in Hungarian: (36) was used. The questionnaire measures general knowledge of diabetes, the nutritional content of foods, the causes of changes in blood sugar levels, and insulin administration. Items were adapted for teachers: items numbered 2, 3, 11, and 12 in the original version were omitted, and additional questions were added based on the findings of Al Duraywish and Nail (38). The final version contained 26 items, each requiring participants to select the one they believed was correct from 3 or 4 response options (e.g., 7. What effect does exercise have on blood glucose levels in a healthy child? a. Decreases, b. Increases, c. No effect). Cronbach’s alpha in our sample was 0.733.

### 2.5 Diabetes attitude scales

#### 2.5.1 DAS3

The items of the Diabetes Attitude Scale (DAS 3) are a general measure of diabetes-related attitudes (34). We adapted some items for teachers on type 1 diabetes: 20 were relevant to diabetes care in schools; therefore, 13 were omitted from the original 33. The instrument contains statements that are phrased as continuations of the sentence “In general, I think that” (e.g., “Teachers should be taught how everyday diabetes care affects the patient’s life”). Participants are asked to indicate on a five-point Likert scale the degree to which they agree with the statements (1**—**Strongly agree, 2**—**Agree, 3**—**Neutral, 4**—**Disagree, 5**—**Strongly disagree). We report additional analysis on the reliability and factor structure of the adapted items in section 2.4, Data Analysis.

#### 2.5.2 School personnel diabetes attitude scale

Based on the publication by Tannous et al. (23), we used 13 items to develop a list measuring the diabetes attitudes of school personnel. The items were statements about diabetes and individuals living with diabetes (e.g., “Children with diabetes should be taught in traditional classes”). Participants indicated their level of agreement with the statements on a 6-point Likert scale (1 = Strongly disagree to 6 = Strongly agree). See the Cronbach alpha values of the subscales used in our study in the results section.

### 2.6 Confidence in diabetes care

For the assessment of confidence in diabetes care, we used 6 statements from the attitude scale developed by Van der Ven et al. (35) (e.g., I know the difference between type 1 and type 2 diabetes), for each of which participants indicated their level of agreement on a 5-point Likert scale (1—strongly disagree—5—strongly agree). Cronbach-*α* in our sample was 0.879.

### 2.7 Data analysis

Statistical analyses were carried out using SPSS software (Version 23). Baseline equivalence between the in-person and online groups was described based on demographic and professional characteristics (Table 1). No formal statistical adjustment for baseline differences was made due to the feasibility-based, non-randomized design and the relatively small sample size. Unless specified otherwise, all analyses involved the full sample of 60 participants. Participants with excessive missing data on a specific scale were excluded from that scale’s analysis. A principal components analysis was conducted to examine the underlying structure of the attitude scales in our sample (DAS3; School personnel diabetes attitude scale). To assess attitude-related changes, we first performed PCA on the adapted attitude scales to identify their factor structure and calculate subscale scores. PCA was performed on the 20 items of the 3-item Diabetes Attitude Scale [DAS3; (34)], adapted for use in school settings. Two subscales emerged. Because the item structure differed from the original scale, the subscales were named accordingly: Social Support (items 4, 6, 18, 19, 20; Cronbach’s *α* = 0.825) and Emotional Effects of Diabetes Care (items 14, 15, 16; Cronbach’s *α* = 0.831). For additional details, see the Appendix. Regarding the School Personnel Diabetes Attitude Scale (23), two participants were excluded due to excessive missing data. PCA was performed on the remaining 13 items, resulting in two subscales: Integration (items 2, 3, 4, 5; Cronbach’s *α* = 0.825) and Distinction (items 10, 11, 13; Cronbach’s *α* = 0.663). Further details are provided in the Appendix. Additional analyses were conducted separately for each attitude questionnaire using the factor scores of the respective subscales. To evaluate the effect of the education, a mixed ANOVA was performed for each scale. *Post hoc* comparisons with Bonferroni correction were used to compare mean scores across the three measurement points.

## 3 Results

### 3.1 Effect of education on diabetes knowledge

One participant’s data were excluded from the present analysis due to too many missing values.

**Figure 3:**
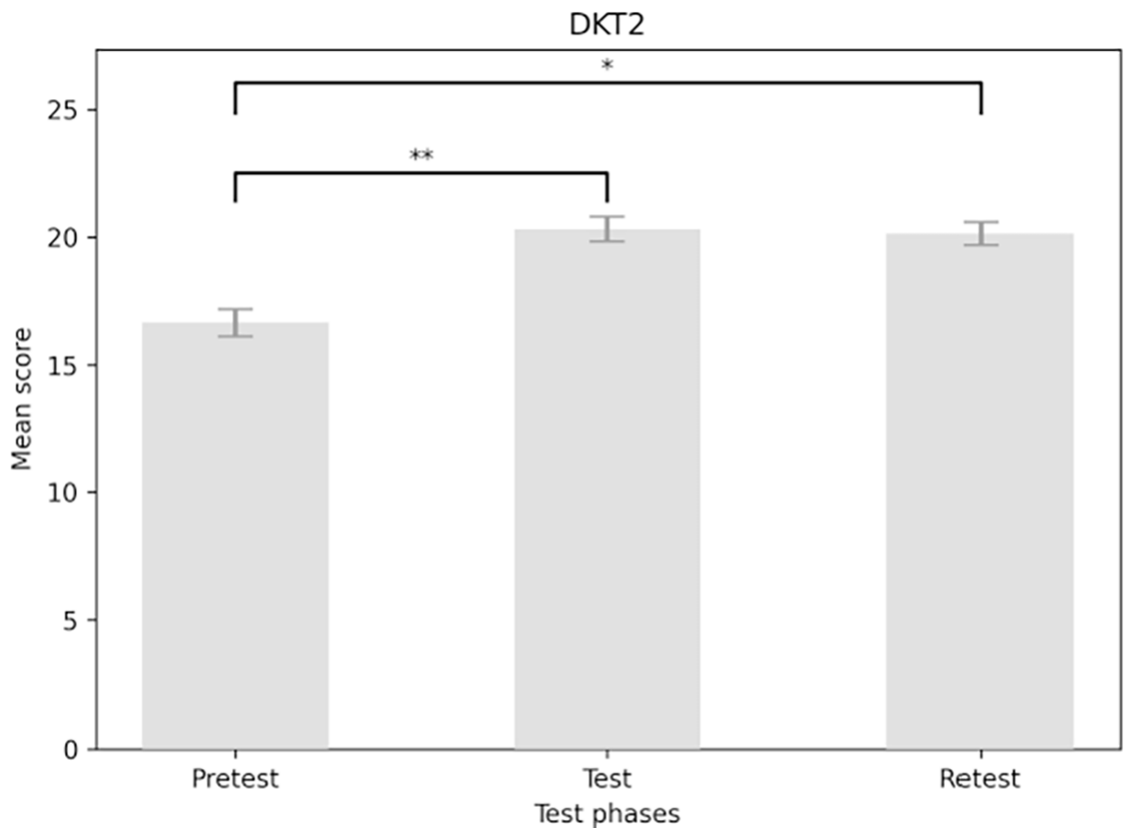
Main effect of DKT2 considering the test phases. DKT2, diabetes knowledge test 2.

**Figure 4:**
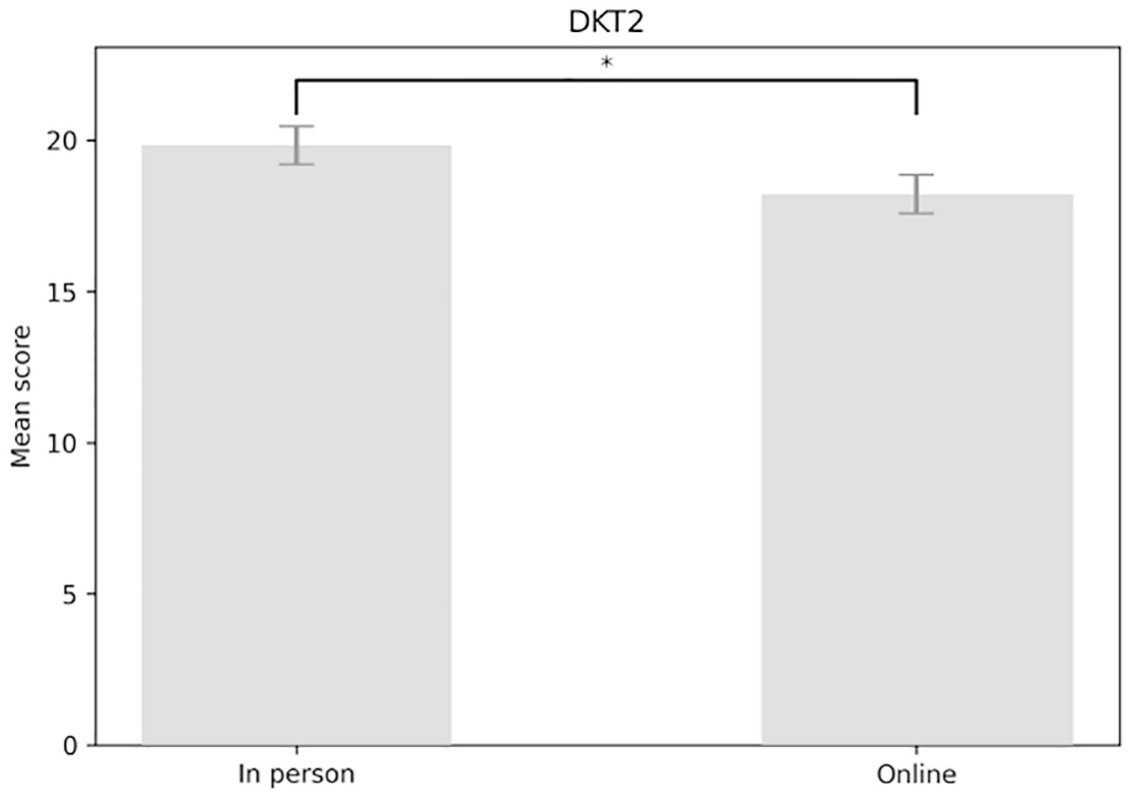
Main effect of the form of education considering DKT2 scores. DKT2, diabetes knowledge test 2.

The analysis revealed a main effect of DKT2 in the expected direction: scores increased significantly from the DKT2 pretest to the test and retest [*F*(1,1.65) = 36.009, *p* < 0.001]. Participants scored higher during the test phase (*M* = 20.315, SE = 0.494) compared to the pretest phase (*M* = 16.641, SE = 0.538) (*p* < 0.001). They also scored higher on the retest (*M* = 20.138, SE = 0.449) than on the pretest (*p* < 0.001) (see Figure 3). The main effect of the form of education was marginally significant [*F*(1,57) = 3.96, *p* = 0.051]. Contrasts indicated that those who received in-person education scored higher (*M* = 19.841, SE = 0.635) on the DKT2 test than those who took it online (*M* = 18.222, SE = 0.508) (see Figure 4).

No significant interaction effect was observed between the DKT score and educational form [*F*(1,1.65) = 2.811, *p* = 0.075].

### 3.2 Impact of education on diabetes attitude

Across all four attitude subscales (Social Support, Emotional Effects of Diabetes Care, Integration, and Distinction), a consistent pattern of results was observed. No significant main effect of measurement occasions (pretest, test, retest) was found in any of the subscales, indicating that scores did not change across measurement occasions regardless of the education modality: *F*(2,116)_Social Support_ = 0.23, *p* = 0.795; F(2,116)_Emotional Effects_ = 0.058, *p* = 0.943; F(2,116)_Integration_ = 0.249, *p* = 0.780; F(2,116)_Distinction_ = 0.092, *p* = 0.912, respectively.

**Figure 5:**
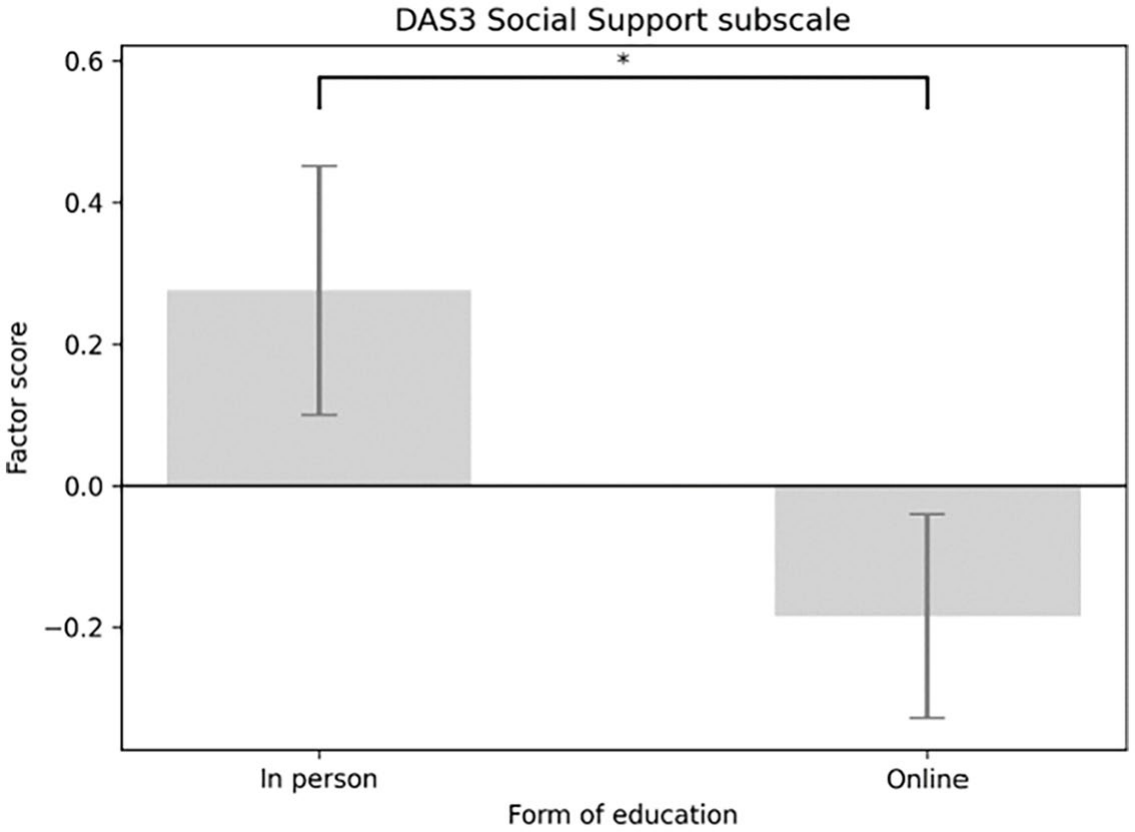
Main effect of the form of education considering DAS3 social support subscale factor scores. DAS3, diabetes attitude scale 3.

**Figure 6:**
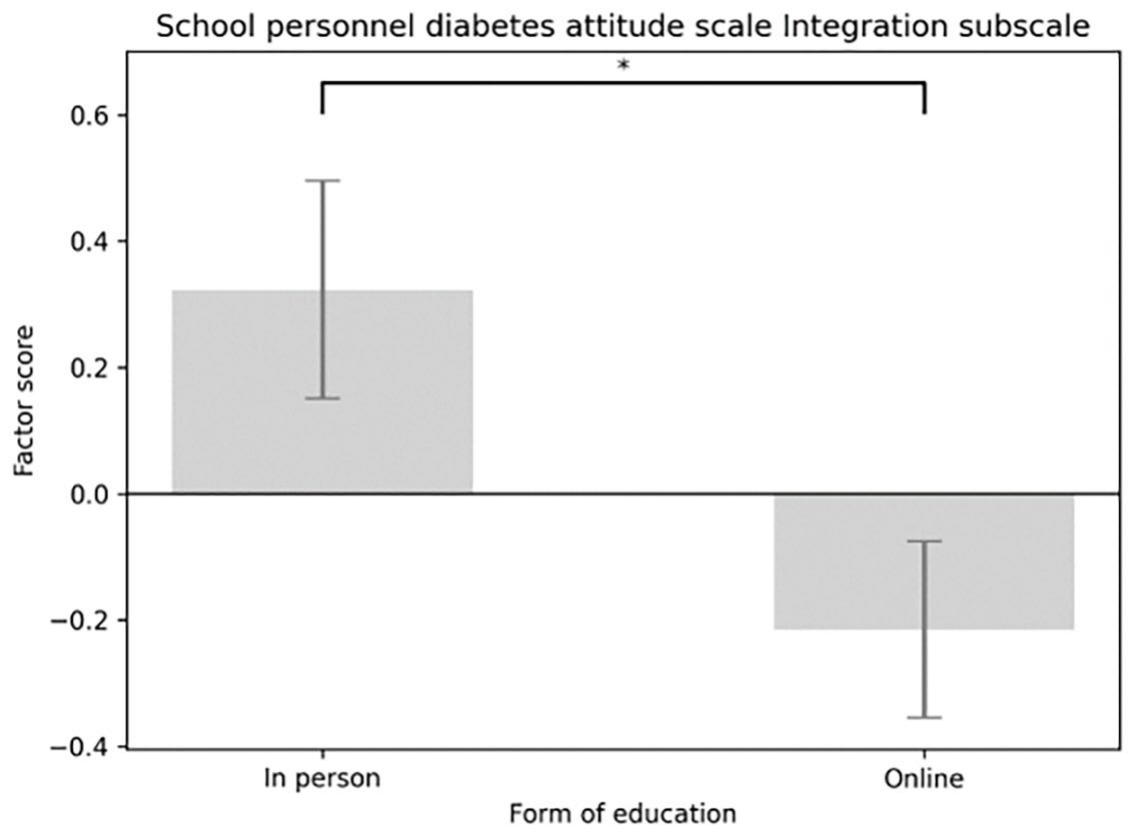
Main effect of the form of education considering School personnel diabetes attitude scale integration subscale factor scores.

**Figure 7:**
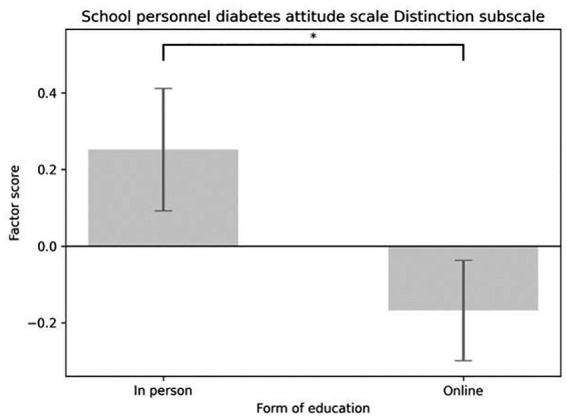
Main effect of the form of education considering school personnel diabetes attitude scale distinction subscale factor scores.

The main effect of the education format (in-person versus online) was significant for three subscales. On the Social Support subscale, participants in the in-person group scored significantly higher than those in the online group [*F*(1,58) = 4.091, *p* = 0.048; *M*_in-person_ = 0.335, SE _in-person_ = 0.176; *M*_online_ = −0.223, SE_online_ = 0.144] (see Figure 5). A similar pattern appeared for the Integration subscale [*F*(1,58) = 5.913, *p* = 0.018; *M*_in-person_ = 0.475, SE_in-person_ = 0.172; *M*_online_ = −0.316, SE_online_ = 0.140] (see Figure 6), and the Distinction subscale [*F*(1,58) = 4.131, *p* = 0.047; *M*_in-person_ = 0.252, SE _in-person_ = 0.160; *M*_online_ = −0.168, SE_online_ = 0.131; see Figure 7]. In contrast, no main effect of education format was observed on the Emotional Effects subscale [*F*(1,58) = 1.963, *p* = 0.167].

**Figure 8:**
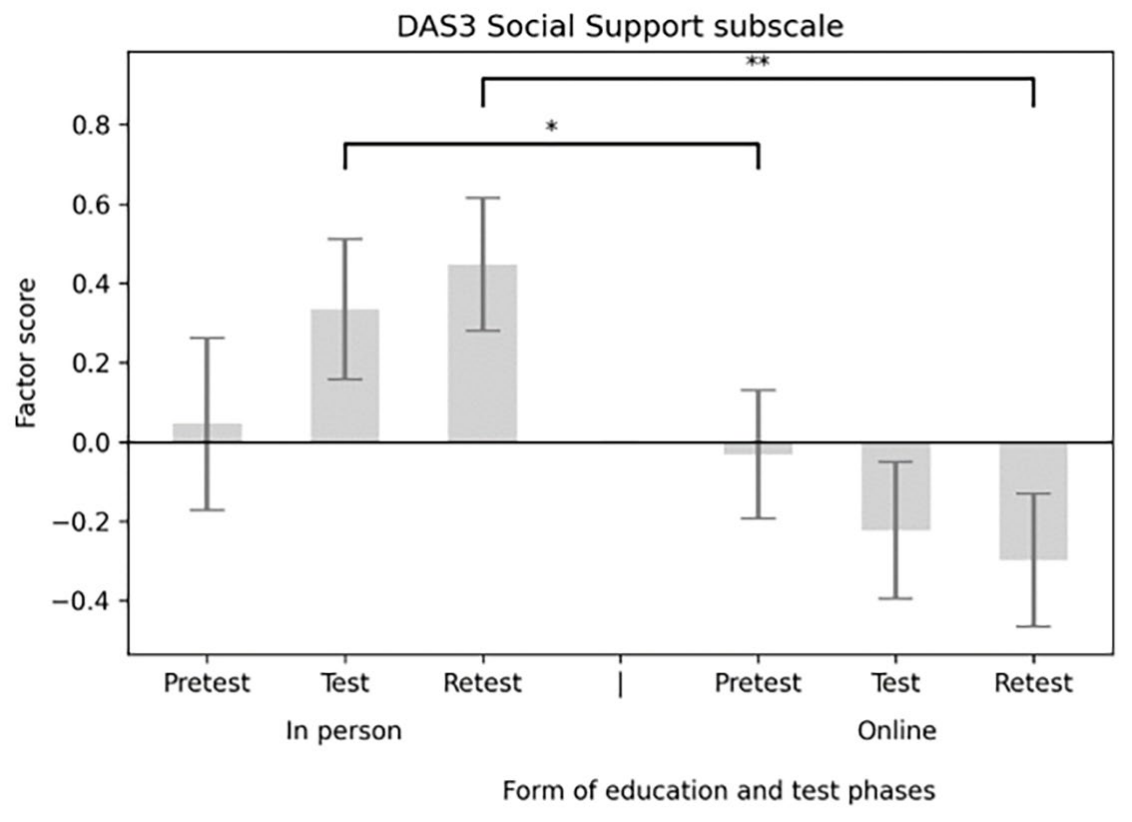
Interaction effect of DAS3 social support scale and the form of education. DAS3, diabetes attitude scale 3.

**Figure 9:**
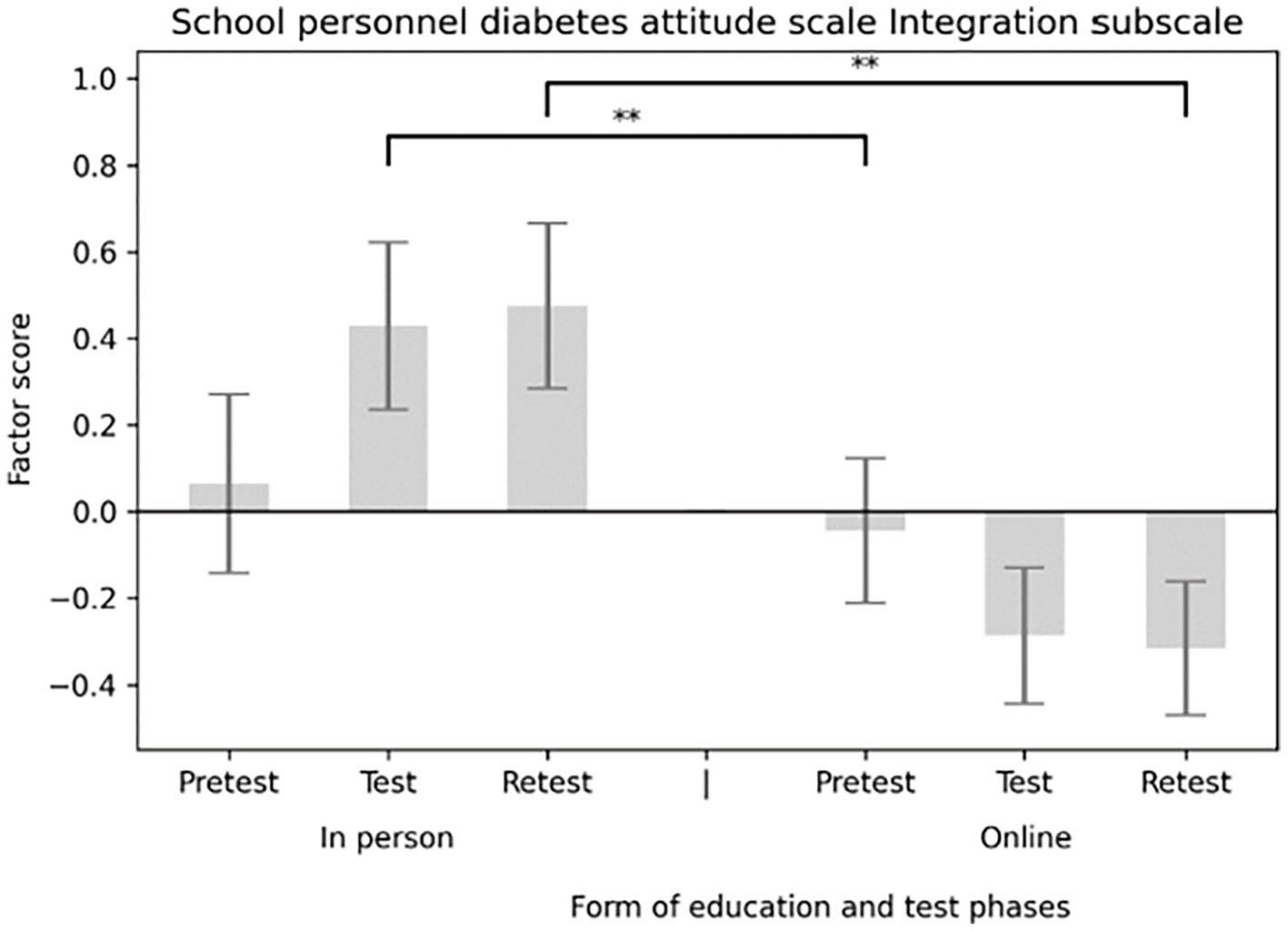
Interaction effect of school personnel diabetes attitude scale integration subscale and the form of education.

To determine whether the effect of the education format varied across different time points, interaction effects between education format and measurement occasion were tested for each subscale. A significant interaction was found between the Social Support subscale and the educational platform [*F*(2,116)_Social Support_ = 0.004, *p* = 0.004]. *Post hoc* tests showed that participants in the in-person condition scored significantly higher both at test (*M*_in-person_ = 0.335, SE_in-person_ = 0.177; *M*_online_ = −0.223, SE_online_ = 0.172; *p* = 0.033) and at retest (*M*_in-person_ = 0.447, SE_in-person_ = 0.167; *M*_online_ = −0.298, SE_online_ = 0.168; p = 0.004) (see Figure 8). A similar pattern appeared for the Integration subscale and the education platform [*F*(2,116)_Integration_ = 6.22, *p* = 0.003], with significantly higher scores in the in-person group at both test (*M*_in-person_ = 0.429, SE_in-person_ = 0.193; *M*_online_ = −0.286, SE_online_ = 0.157; *p* = 0.006) and retest (*M*_in-person_ = 0.475, SE_in-person_ = 0.190; *M*_online_ = −0.316, SE_online_ = 0.155; *p* = 0.002) (see Figure 9). No significant interaction effects were observed for the Distinction [*F*(2,116)_Distinction_ = 2.297, *p* = 0.105] or Emotional Effects subscales [*F*(2,116)_Emotional Effects_ = 1.460, *p* = 0.236].

### 3.3 Confidence in diabetes care scale

**Figure 10:**
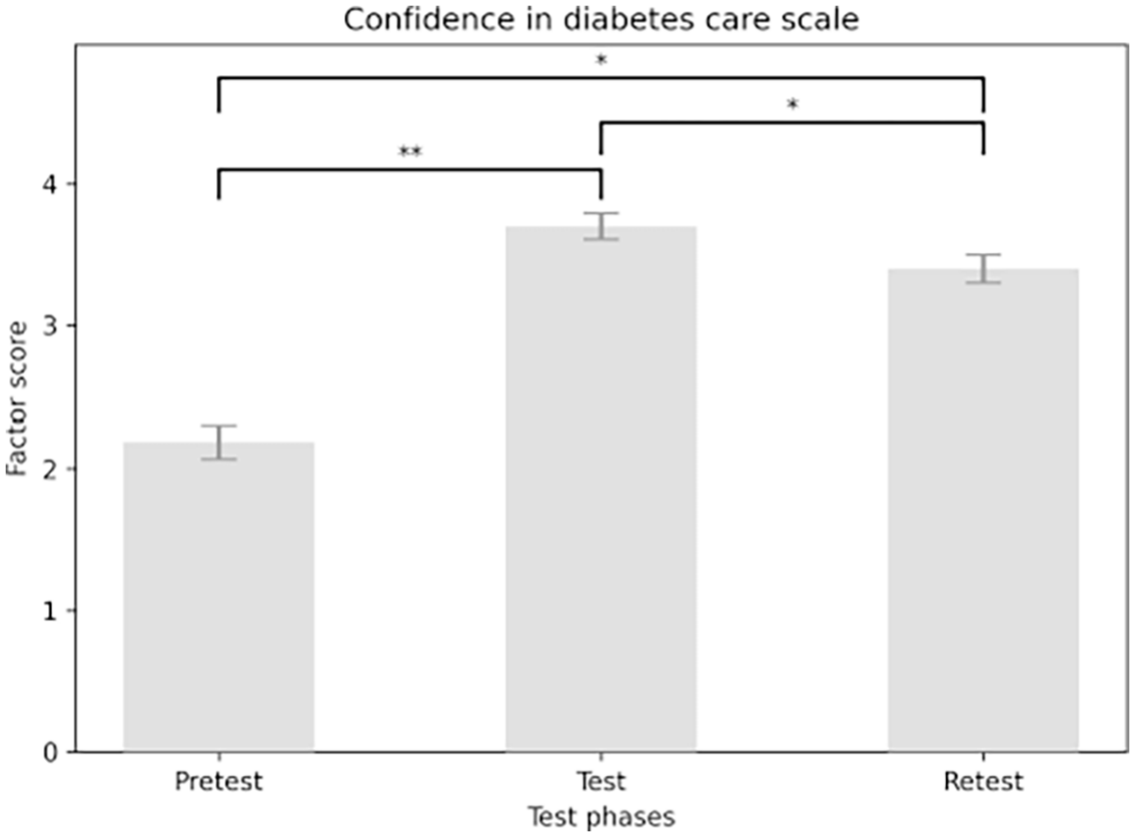
Main effect of confidence in diabetes care scale considering the test phases.

**Figure 11:**
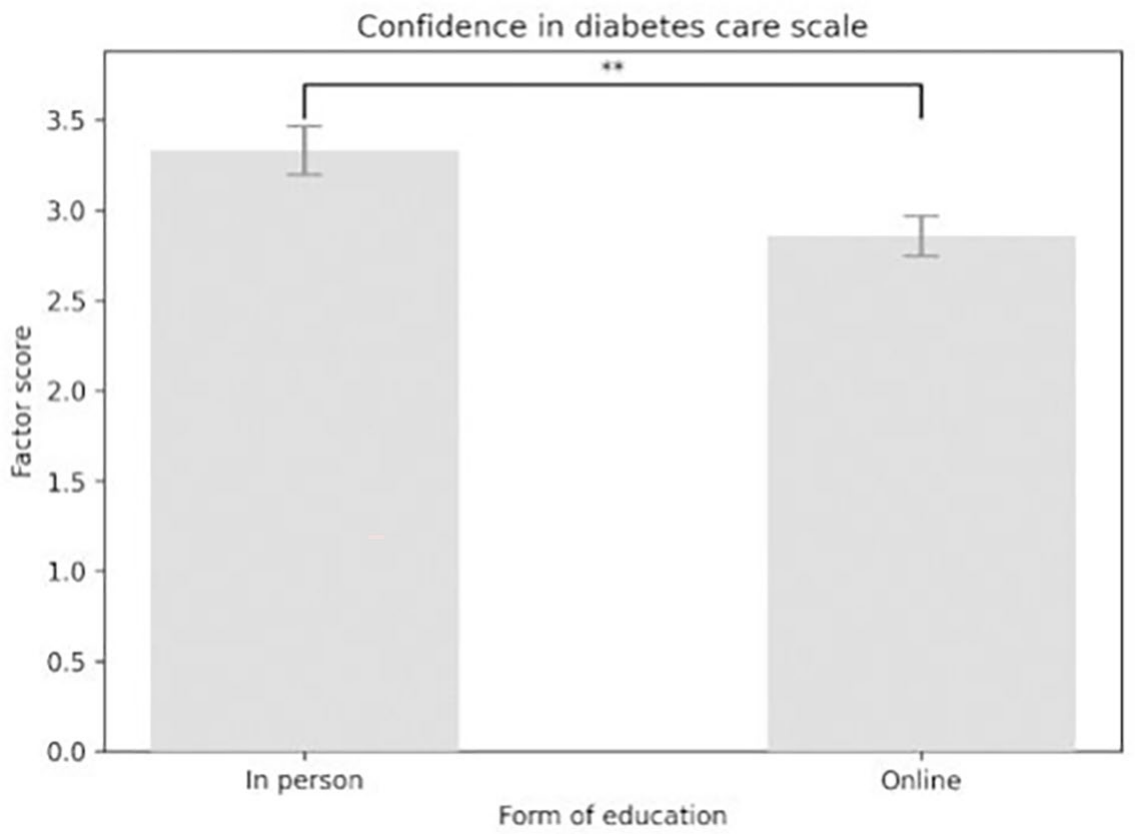
Main effect of the form of education considering confidence in diabetes care scale scores.

The analysis showed a main effect of the Confidence in Diabetes Care scale in the expected direction: there was a significant increase in diabetes confidence scores from pretest to test and retest [*F*(2,116) = 131.441, *p* < 0.001]. Participants scored higher on the test (*M* = 3.698, SE = 0.094) than on the pretest (*M* = 2.186, SE = 0.118) (*p* < 0.001). They also scored higher on the test than on the retest (*M* = 3.398, SE = 0.098) (*p* = 0.005). Additionally, scores on the retest (*M* = 3.398, SE = 0.098) were higher than on the pretest (*p* < 0.001) (see Figure 10). The main effect of educational format was significant [*F*(1, 58) = 7.597, *p* = 0.008]. Contrasts indicated that those who received in-person education scored higher (*M* = 3.333, SE = 0.134) on the diabetes confidence scale than those who took it online (*M* = 2.855, SE = 0.11) (see Figure 11). No significant interaction effect was found between the scale score and the education platform [*F*(2,116) = 1.525, *p* = 0.222].

## 4 Discussion

In this study, we examined whether a short, standardized diabetes education lecture could boost diabetes knowledge and enhance attitudes and confidence in diabetes care. We also analyzed the differences between in-person and online effects. The intervention was successfully carried out in both formats, and all planned educational sessions were delivered. The intervention was not explicitly grounded in theory; however, the results suggest that increased knowledge and hands-on experience may have improved confidence. The absence of immediate post-test and day 30 attitude changes shows that more intensive or experiential components might be necessary to influence attitudes.

The results of the intervention showed a significant increase in diabetes knowledge from the pretest to the test and from the pretest to the retest. These findings suggest that the intervention effectively enhanced participants’ diabetes knowledge, which was retained for 30 days. These significant increases were observed both online and in person. However, teachers who participated in person had slightly higher knowledge scores than those who participated online. Similar improvements in teachers’ diabetes knowledge have been reported in recent school-based interventions, including a quasi-experimental study in Turkey (29) and a large-scale intervention involving more than 300 teachers in Morocco (30).

The Emotional Effects of Diabetes Care subscale of DAS3 assessed how frustrating participants found diabetes care for children with T1D, for parents, and for teachers. The Social Support subscale measured how important they believed supporting the social network of the child with diabetes was, and whether teachers should be educated about diabetes and be involved in blood glucose management. The Integration subscale of the School Personnel Diabetes Attitude Scale (23) addressed the integration of children into society and groups. The Distinction subscale included items on discrimination against children with diabetes and disadvantages in school due to the disease (such as increased absenteeism and its impact on learning skills). None of the subscale scores showed significant improvement immediately post-test or at day 30. However, a significant difference was observed between the online and in-person groups’ scores. Interestingly, scores trended to increase after in-person training and decreased after online training. For the distinction subscale, this suggests that online participants exhibited less discrimination after the education than in-person participants. In contrast to our findings, Bechara et al. (19) reported substantial changes in school staff attitudes following the KiDS intervention, suggesting that longer, more comprehensive programs may be more effective at promoting attitude change than brief, single-session education.

The lack of significant change in attitude scores may be interpreted in light of established psychological theories of attitude formation and change. Attitudes are commonly conceptualized as multidimensional constructs comprising cognitive, affective, and behavioral components (39–41). Educational interventions can readily influence the cognitive component by increasing knowledge and awareness; however, changes in affective responses and behavioral orientations are typically more gradual and are shaped by prior experiences and existing belief structures (40). Brief lecture-based formats primarily target cognitive understanding and may provide limited opportunities for affective or experiential engagement, which are often necessary for shifts in deeper evaluative orientations (42).

According to Petty and Cacioppo (43) the integration of new knowledge into stable attitudinal dispositions requires deeper cognitive elaboration and personal relevance, processes that are more likely to occur when individuals actively engage with and reflect on new information. When such elaborative processing and experiential anchoring are limited, newly acquired knowledge may remain primarily cognitive and may not translate into measurable changes in evaluative orientations (43). This could be the case related to inclusion, discrimination, or the everyday challenges faced by children with type 1 diabetes. Accordingly, the absence of significant short-term changes in Distinction and Integration attitudes observed in the present study may reflect the relative stability of attitudinal constructs (40) and the limited time frame and informational nature of the intervention.

As several studies have shown, higher levels of diabetes knowledge are linked to more positive attitudes toward diabetes (13, 22, 23). In our study, knowledge levels increased significantly, but there was no notable change in most attitude scores after the education. From a health literacy perspective, educational interventions mainly aim to improve knowledge and perceived competence, while changes in attitudes and behavior generally require more complex and sustained learning experiences (44, 45). Further investigation is needed to clarify the relationship between knowledge and attitude in diabetes interventions.

The items of Confidence in diabetes care included diabetes competence, recognition of hypoglycemia and hyperglycemia, and the ability to provide appropriate diabetes management. Results showed significant improvements in confidence related to diabetes care across all three measurement sessions. Similar findings were reported by Taha et al. (25) and Gutierrez (26). Dixe et al. (27) also found that diabetes education increased both diabetes knowledge and confidence in diabetes management. The effectiveness of an online program was supported by Bassi et al. (31). In this study, in-person education yielded higher mean scores than online education. Our findings suggest that in-person education might be more effective at boosting confidence in diabetes care than online platforms. This confirms the importance of interactivity and multimedia tools in online education (26).

## 5 Limitations

A key limitation of our study is the relatively small sample size; therefore, the generalizability of the findings should be interpreted with caution. However, as noted by Cohen et al. (46) and Gall et al. (47), a minimum of 15 participants per group is considered sufficient for comparative analysis. The baseline characteristics of the participants were comparable to those typically reported for practicing kindergarten and school teachers. Another limitation is the lack of control over how participants engaged with the online educational material. Although we provided detailed instructions and asked participants to follow the steps, we could not verify whether the material was fully and properly completed. Consequently, the comparison between online and in-person formats lacks full objectivity. The generalizability of the findings is further limited by the short follow-up period, especially regarding attitude change and the specific educational context in which the intervention was implemented. Finally, most of the questionnaires used were not originally validated for our specific target group (teachers); although we performed statistical verification, this remains a methodological limitation.

## 6 Conclusion

Overall, the current findings show that a short, one-hour diabetes education session can positively influence teachers’ knowledge about diabetes and their confidence in managing it, with these effects lasting at least 1 month. However, this did not significantly change attitudes related to diabetes, indicating that more engaging, hands-on, and experience-based educational methods might be necessary to affect deeper attitudes. To foster meaningful attitude shifts, future programs could focus more on raising awareness and providing insight into the daily experiences of children and families living with type 1 diabetes. These findings align with earlier school-based educational efforts that demonstrate short training sessions mainly boost knowledge and perceived confidence (19, 25, 26, 31), which are vital components of health literacy (44, 45). In our study, face-to-face education proved more effective than other formats, especially in maintaining confidence over time. Considering the rising number of children with type 1 diabetes in Europe (1) and the limited access to ongoing healthcare support in schools (7, 8), implementing brief, standardized diabetes training for teachers could be a practical and cost-efficient way to improve school readiness and create safer, more supportive learning environments for children with type 1 diabetes.

## Data Availability

The raw data supporting the conclusions of this article will be made available by the authors, without undue reservation.

## Appendix

### Principal component analysis of the diabetes attitude scale (DAS3)

According to the *KMO-test* (0.714), the data were suitable for factor analysis. The variables with less than 0.4 extraction communalities were excluded; therefore, the final analysis was performed, including eight items. We identified two components with eigenvalues of 3.087 and 2.425, respectively (all the other emerging components having eigenvalues less than 1). The two components accounted for 68.903 percent of the variability. Two subscales were found. As the items belonging to the subscales differed from the ones of the original scale, we named the subscales as follows: Social support subscale: items 4, 6, 18, 19, and 20 (Cronbach-*α*: 0.825); and Emotional effects of diabetes care subscale: items 14, 15, 16 (Cronbach-*α*: 0.831). According to the Bartlett’s sphericity test [*χ^2^*(28) = 212.825, *p* < 0.001], these eight items were related to each other.

### Principal component analysis of the school personnel diabetes attitude scale

According to the *KMO-test* (0.759), the data are suitable for factor analysis. The variables with less than 0.4 extraction communalities were excluded; therefore, the final analysis was performed, including seven items. We identified two components with eigenvalues of 2.888 and 1.654, respectively (all the other emerging components having eigenvalues less than 1). The two components accounted for 68.903 percent of the variability. Two subscales were found: the integration subscale with items 2, 3, 4, and 5 (Cronbach-*α*: 0.825) and the distinction subscale with items 10, 11, and 13 (Cronbach-α: 0.663). According to the Bartlett’s sphericity test [*χ^2^*(21) = 123.387, *p* < 0.001], these seven items were related to each other (23).
